# Supplementary material for: Time Estimation Following an Exhaustive Exercise
Source: J Funct Morphol Kinesiol. 2025 Jan 16;10(1):35. doi: 10.3390/jfmk10010035 (PMC11755588; doi:10.3390/jfmk10010035)
Supplement: Supplementary file 1 [file jfmk-10-00035-s001.zip › jfmk-3421541-supplementary.pdf]

Figure	Mean	S.D.	Statistical Method	# samples	Comparison	P value	Summary
**Fig. 2 left - Blood lactate levels in mental count**							
W pre	1.567	0.2498	ANOVA; Kruskal-Wallis test followed by Dunn's Multiple Comparison Test.	12	W pre vs. W end	<0.0001	****
W end	7.167	1.002		12	W pre vs. W 5 min	0.0113	*
W 5 min	6.25	0.9539		12	W pre vs. W 15 min	>0.9999	ns
W 15 min	1.583	0.229		12	W end vs. W 5 min	>0.9999	ns
					W end vs. W 15 min	0.0002	***
					W 5 min vs. W 15 min	0.0254	*
M pre	1.633	0.2605		12	M pre vs. M end	<0.0001	****
M end	7.825	0.5578		12	M pre vs. M 5 min	0.0203	*
M 5 min	6.592	0.3895		12	M pre vs. M 15 min	>0.9999	ns
M 15 min	1.6	0.1537		12	M end vs. M 5 min	>0.9999	ns
					M end vs. M 15 min	<0.0001	****
					M 5 min vs. M 15 min	0.0172	*
**Fig 2 right Blood lactate levels in non-mental count**							
W pre	1.6	0.2045	ANOVA; Kruskal-Wallis test followed by Dunn's Multiple Comparison Test.	12	W pre vs. W end	0.0067	****
W end	7.1	0.7663		12	W pre vs. W 5 min	>0.9999	**
W 5 min	6.433	0.514		12	W pre vs. W 15 min	>0.9999	ns
W 15 min	1.658	0.1929		12	W end vs. W 5 min	>0.9999	ns
					W end vs. W 15 min	0.0001	***
					W 5 min vs. W 15 min	0.0247	*
M pre	1.592	0.2193		12	M pre vs. M end	<0.0001	****
M end	7.258	0.8522		12	M pre vs. M 5 min	0.0023	**
M 5 min	6.525	0.5545		12	M pre vs. M 15 min	>0.9999	ns
M 15 min	1.658	0.1929		12	M end vs. M 5 min	>0.9999	ns
					M end vs. M 15 min	<0.0001	****
					M 5 min vs. M 15 min	0.0099	**
**Time overestimation (%) in mental count**							
W pre	84.53	34.03	ANOVA; Kruskal-Wallis test followed by Dunn's Multiple Comparison Test.	12	W pre vs. W end	0.0214	*
W end	22.62	17.6		12	W pre vs. M pre	>0.9999	ns
					W pre vs. M end	0.6427	ns
					W pre vs. All pre	>0.9999	ns
M pre	78.61	14.25		12	W pre vs. All end	0.0391	*
M end	24.06	9.417		12	W end vs. M pre	0.0002	***
					W end vs. M end	>0.9999	ns
					W end vs. All pre	0.0002	***
All pre	81.57	25.69		24	W end vs. All end	>0.9999	ns
All end	23.34	13.83		24	M pre vs. M end	0.0193	*
					M pre vs. All pre	>0.9999	ns
					M pre vs. All end	0.0002	***
					M end vs. All pre	0.037	*
					M end vs. All end	>0.9999	ns
					All pre vs. All end	<0.0001	****

Number of correct answers in non-mental count							
W pre	8.833	0.8348	ANOVA; Kruskal-Wallis test followed by Dunn's Multiple Comparison Test.	12	W pre vs. W end	0.0004	***
W end	7.083	1.24		12	W pre vs. M pre	>0.9999	ns
					W pre vs. M end	0.0012	**
					W pre vs. All pre	>0.9999	ns
M pre	9.333	0.6513		12	W pre vs. All end	<0.0001	****
M end	7.833	1.03		12	W end vs. M pre	0.0002	***
					W end vs. M end	>0.9999	ns
					W end vs. All pre	<0.0001	****
All pre	81.57	25.69		24	W end vs. All end	>0.9999	ns
All end	23.34	13.83			M pre vs. M end	0.0006	***
					M pre vs. All pre	>0.9999	ns
					M pre vs. All end	<0.0001	****
					M end vs. All pre	<0.0001	****
					M end vs. All end	>0.9999	ns
					All pre vs. All end	<0.0001	****

CORRELATIONS

	R squared	F	P value	Summary	Equation	# samples
**Fig 3. Time overestimation (%)-blood lactate level: mental count**						
Women	0.5549	27.43	<0.0001	****	Y = -10.42*X + 99.09	24
Men	0.8101	93.83	<0.0001	****	Y = -8.536*X + 91.70	24
All	0.6379	81.04	<0.0001	****	Y = -9.394*X + 95.18	48
**Fig 4. Corrects answers (#)-blood lactate level: non-mental count**						
Women	0.4279	16.45	0.0005	***	Y = -0.3123*X + 9.317	24
Men	0.3831	13.66	0.0013	**	Y = -0.2383*X + 9.638	24
All	0.3093	23.28	<0.0001	****	Y = -0.2466*X + 9.284	48
**Fig. 5. Difference in duration (%)- incidence of errors (%): non-mental count**						
Diff (%) pre	0.3179	3.728	0.0896	ns	Y = -0.2103*X + 32.09	10
Diff (%) end	0.4494	6.530	0.0339	*	Y = -0.2103*X + 32.09	10
